# Longitudinal Average Glucose Levels and Variance and Risk of Stroke: A Chinese Cohort Study

**DOI:** 10.1155/2020/8953058

**Published:** 2020-04-21

**Authors:** Xuenan Peng, Jinzhuo Ge, Congju Wang, Hongpeng Sun, Qinghua Ma, Yong Xu, Yana Ma

**Affiliations:** ^1^Medical College of Soochow University, Suzhou 215123, China; ^2^Department of Child Health, Jiangsu Key Laboratory of Preventive and Translational Medicine for Geriatric Diseases, School of Public Health, Soochow University, Suzhou 215123, China; ^3^Centers for Disease Control and Prevention of Suzhou High-Tech Zone, Suzhou 215000, China; ^4^The 3rd People's Hospital of Xiangcheng District, Suzhou 215134, China

## Abstract

**Background:**

Diabetes is a known independent risk factor for stroke. However, whether higher glucose levels (126–139.9 mg/dl) can increase the risk of stroke in people without diabetes is still unknown. Moreover, as a fluctuating parameter, long-term glucose levels may also be related to the risk of stroke outcome. It is important to explore the correlation between long-term average blood glucose, as well as its variability, and stroke.

**Methods:**

We used 40,975 clinical measurements of glucose levels and 367 measurements of glycated hemoglobin A1c levels from 12,321 participants without stroke to examine the relationship between glucose levels and the risk of stroke. Participants were from the Weitang Geriatric Diseases study, including 5,707 men and 6,614 women whose mean age at baseline was 60.8 years; 1,011 participants had diabetes, and 11,310 did not. We estimated the long-term average blood glucose level based on the multilevel Bayesian model and fit in Cox regression models, stratified according to diabetes status.

**Results:**

Over a median follow-up period of 5 years, stroke developed in 279 of the 12,321 participants (244 without diabetes and 35 with). For people with an average glucose level of 126–139.9 mg per deciliter, compared with 90–99.9 mg per deciliter, the adjusted hazard ratio (HR) for total stroke was 1.78 (95% confidence interval (CI), 1.16–2.75), and the HR for levels higher than 140 mg per deciliter was 1.89 (95% CI, 1.09–3.29). Among those without diabetes whose glucose level was higher than 140 mg per deciliter, compared with 90–99.9 mg per deciliter, the adjusted HRs for total stroke and fatal stroke were 3.66 (95% CI, 1.47–9.08) and 5 (95% CI, 1.77–14.15), respectively. For a glucose standard deviation level higher than 13.83 mg per deciliter, compared with that lower than 5.91 mg per deciliter, the adjusted HR for total stroke was 2.31 (95% CI, 1.19–4.48).

**Conclusions:**

Our results suggest that higher average glucose levels (126–139.9 mg/dl) and variance may be risk factors for stroke, even among people without diabetes diagnosis.

## 1. Introduction

Stroke is the third leading cause of death in the world, seriously threatening human health and placing economic and medical burden on society and families [1]. Diabetes mellitus (DM) is a known risk factor for stroke, and hyperglycemia can cause various microvascular and macrovascular diseases [1, 2]. However, the development of diagnostic criteria for diabetes is based on the risk of developing eye and kidney disease rather than cardiovascular disease [3]. Some studies have shown that high fasting plasma glucose (FPG) levels were significantly associated with a subsequent risk of cardiovascular disease in individuals without diabetes [4–6], so it is very likely that blood glucose levels below the diagnostic criteria will also cause cardiovascular disease or that there is no threshold for FPG and cardiovascular disease risk [7]. Some studies have found that the risk of cardiovascular disease is directly proportional to the concentration of FPG [3], while other studies found that the relationship between the two is J-shaped [8].

Moreover, people's blood glucose levels fluctuate, and one blood glucose measurement cannot accurately represent long-term average FPG levels. Therefore, it is important to explore the correlation between long-term average blood glucose and the risk of stroke, rather than random FPG measurements. In addition, some studies have found that the rate of blood glucose variability is related to the risk of kidney disease, retinopathy, and cardiovascular disease because FPG fluctuation may cause a series of injury responses, such as reactive oxygen production, inflammation, and endothelial dysfunction [9]. Therefore, it is also important to investigate the association between blood glucose variability and stroke.

In previous studies, the relationship between blood glucose and the risk of cardiovascular disease was measured by only one or a few blood glucose value measurements, which may lead to bias and the misinterpretation of the correlation [6, 10, 11]. Therefore, in our study, we estimated long-term average blood glucose based on a multilevel Bayesian model.

## 2. Research Design and Methods

### 2.1. Study Population

The Weitang Geriatric Diseases study was a community-based survey conducted in Weitang, an urban metropolis located in Suzhou in eastern China. This study initially included 12,413 randomly selected members of a preventive medical examination. All members were stroke-free and had no previous stroke history. From 2011 to 2012, participants aged 45 years old or older at the time of enrollment were included. Participants were invited to return at 1-year intervals for the purpose of collecting FPG data and identifying incident cases of stroke and other chronic diseases by conducting annual physical examinations. The sample for the current study was limited to 12,321 participants who had at least one follow-up visit and had at least one measurement of glucose or glycated hemoglobin A1c (HbA1c). The study procedures were approved by the Institutional Review Board of Soochow University, and all the participants provided their written informed consent.

### 2.2. Stroke Outcomes Assessment

The outcome was the first occurrence of stroke, either nonfatal or fatal. The incidence date of stroke was recorded when stroke was diagnosed in the hospital. All potential stroke cases were identified by the stroke code (tenth versions of International Classification of Diseases, I63), death certificates, and questionnaires. Two physicians who were experienced neurologists and blinded to the FBG concentrations reviewed the medical records and judged the cases annually. In cases of disagreement, a third physician was consulted to make the final decision. Study participants were assessed for stroke every year with the use of a regional health information system. Stroke-free participants continued with scheduled follow-up visits. Fatal cases were confirmed by medical records, autopsy reports, or death certificates with stroke as the primary cause. Nonfatal stroke was defined as the sudden onset of a focal neurological deficit and the demonstration of acute primary intraparenchymal hemorrhage on either brain computed tomography or magnetic resonance imaging, which was available for all suspected nonfatal stroke cases.

### 2.3. Assessment of FPG

Participants were classified as having diabetes (with previous hospital diagnosis certificate) or not having diabetes (including undiagnosed diabetes) at enrollment according to their previous medical history and a personal statement. FPG or glycated hemoglobin levels for each participant were collected via at least one measurement during the follow-up years. Fasting plasma samples were collected in the morning after an 8 h overnight fast and were transfused into vacuum tubes containing EDTA. Plasma was separated from blood immediately and stored at 4°C. Blood glucose concentrations were measured 4 h after blood sample collection. The collection was divided into two samples used to test FPG and HbA1c. Blood glucose was determined by Glucose Oxidase method (GOD-POD) using Roche Cobas 501 automatic biochemical analyzer, and HbA1c was analyzed by high-performance liquid chromatography method with TOSOH HLC-723G7 automatic glycohemoglobin analyzer. Once nondiabetic participants were diagnosed with diabetes during the follow-up period, they would be divided into diabetic groups. For those whose FPG exceeded 126 mg/dl, as well as those who were not diagnosed by a a physician, their group status remained unchanged as undiagnosed diabetes. Average glucose levels were categorized into 6 ranges (80–89.9, 90–99.9, 100–109.9, 110–125.9, 126–139.9, and >140 mg/dL).

We selected an analytical strategy using a hierarchical Bayesian framework [12] to develop a measure of average glucose levels in the prior 5 years based on clinical laboratory measures of glucose and HbA1c for all time frames in which at least one measurement of glucose or HbA1c was available. We transformed the calculated HbA1c values to daily average glucose values with this formula: daily average glucose = (28.7 × HbA1c) − 46.7, while stabilizing estimates for individuals with relatively sparse observations by borrowing information across participants with more measurements, and accounting for the relative variability in glucose and HbA1c measures by creating a combined estimate. Let *X*_*ij*_ represent the *j*th glucose measures for the *i*th participant, and let *Y*_*ij*_ represent the *j*th measure of glucose in the time period of interest estimated from HbA1c for participant *i*. We assume the hierarchical model *X*_*ij*_ ~ *N*(*μ*_*i*_, *σ*_*X*_^2^), *Y*_*ij*_ ~ *N*(*μ*_*i*_, *σ*_*Y*_^2^), *μ*_*i*_ ~ *N*(*θ*, *τ*^2^), where *μ*_*i*_ represents the average daily glucose level for the *i*th individual in the time period of interest. Then an empirical Bayes estimation formula was used to calculate *μ* from *X* and *Y*. This model assumes that given a participant's average daily glucose level, measured glucose and glucose estimated from HbA1c are independent and vary randomly around an individual's daily average with variance *σ*_*X*_^2^ and *σ*_*Y*_^2^, respectively, and that, in the population of interest, average daily glucose levels vary across individuals with population mean *θ* and variance *τ*^2^. In this framework, *θ* means prior or population mean average glucose which is the data based on clinical parameters of large population.

### 2.4. Assessment of Covariates

The baseline assessment, which was performed after 8 h of overnight fasting, included a physical examination; clinical evaluations such as blood chemistry analyses and blood pressure measurement; questionnaires on personal and family medical history, smoking habits, and drinking habits; and an exercise test. Briefly, body mass index (BMI) was calculated from measured weight and height (kg/m^2^); alcohol consumption was quantified as drinks per week (drinks/week); and smoking status included never, former smoker, or current smoker. We defined current smoking as smoking more than one cigarette per day for more than one year, and former smoking as stopping smoking for at least one year since enrollment and having smoking history in the past. Exercise level was assessed with questions about types of physical activity (sedentary or active) and the number of times exercise was performed in a week. Those who exercised 3 or more days per week were categorized as having regular exercise. Blood pressure was determined by averaging two measurements on the right arm while the participant was seated, with a 5-minute rest period between each measurement. Hypertension was defined as either systolic/diastolic blood pressure of 140/90 mmHg or higher or a history of hypertension. Hypercholesterolemia was defined as serum total cholesterol of 240 mg/dL or higher. Serum samples were analyzed in the laboratory of the 3rd People's Hospital of Xiangcheng District, Suzhou.

### 2.5. Statistical Analysis

The average blood glucose of the participants estimated by the Bayesian method was divided into six groups according to the average blood sugar concentration. The Cox regression model was used to assess the relationship between blood glucose and the risk of stroke. First, hazard ratios (HRs) and the 95% confidence interval (CI) for the relationship between FPG and the risk of stroke in all participants with and without diabetes were calculated by Cox regression model. There are three Cox regression models. The independent variable in model 1 is average blood glucose, and model 2 adjusts for age and gender. Model 3 includes the factors used in model 2 in addition to education, smoking behavior, drinking behavior, regular exercise, BMI, systolic blood pressure, and total cholesterol. Second, we predicted the linear trend between blood glucose levels and risk of stroke in different groups. Glucose levels were incorporated in models with the use of natural cubic splines with 4 knots (at the 5th, 35th, 65th, and 95th percentiles) to allow for a nonlinear association between glycemia and risk of stroke as measured by the log hazard [13]. Third, we calculated the degree of blood glucose variability (standard deviation, SD) in participants with no less than twice follow-up data based on the Bayesian method and determined the quartiles according to the division of the standard deviation into four groups, repeating the above two steps to explore the risk of stroke. All *P* values were 2-tailed, and a significance level of 0.05 was used. Statistical analysis was conducted using SAS statistical software, version 9.4 (SAS Institute Inc., Cary, NC).

## 3. Results

The baseline characteristics of the 12,321 study participants are presented in Table 1. Upon enrollment, the average age of study participants was 60.8 years for participants without diabetes and 61.3 years for participants with diabetes, and the average BMI was 23.5 kg/m^2^ and 24.8 kg/m^2^ for each group, respectively. The average fasting glucose values were 102.5 mg/dL and 154 mg/dL in those without and with diabetes, respectively. In the normal group, 24.9% were current smokers, and 67.1% had never been smokers; among the DM group, the corresponding figures were 23.2% and 68.6%, respectively. There are 40,975 values available for fasting glucose levels and 367 values available for HbA1c levels.

Over a median follow-up period of 5 years, stroke developed in 279 of the 12,321 participants (2.26%), including 244 of the 11,310 participants who did not have diabetes at the end of follow-up (2.2%) and 35 of the 1,011 participants who had stroke at the end of follow-up (3.5%). A total of 133 participants had probable or possible nonfatal stroke at the end of follow-up, and 146 had fatal stroke.

**Figure 1:**
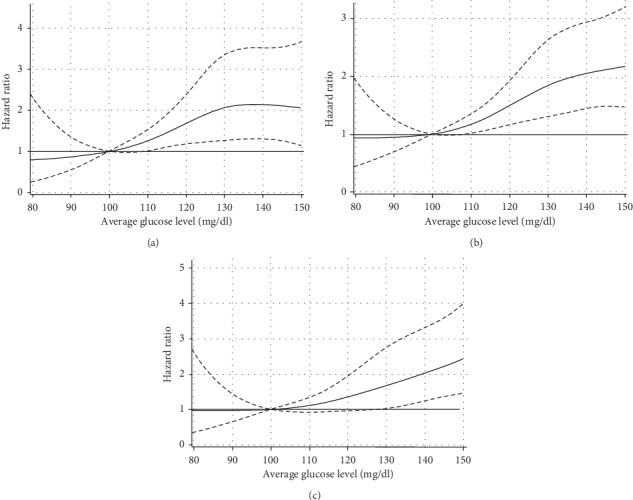
Risk of incident stroke associated with the average glucose level during the preceding 5 years.

Associations between average glucose levels and the development of stroke are shown in Table 2. Among participants, an increased risk of total stroke with increasing glucose levels was significant (*P*=0.0009) for model 3, and restricted cubic spline regression models showed that lower limits of 95% CI for HR are more than 1.0 when average glucose level was higher than 110 mg per deciliter (Figure 1). For an average glucose level of 126–139.9 mg per deciliter compared with 90–99.9 mg per deciliter, the adjusted HR for total stroke was 1.78 (95% CI, 1.16–2.75), and the HR for more than 140 mg per deciliter was 1.89 (95% CI, 1.09–3.29). Those with higher levels of glucose had an increased risk of nonfatal stroke (*P*=0.0067). For an average glucose level of 126–139.9 mg per deciliter compared with 90–99.9 mg per deciliter, the adjusted hazard ratio for nonfatal stroke was 2.16 (95% CI, 1.19–3.93). The association of glycemia with risk of stroke was similar across subgroups stratified according to sex and history of hypertension (*P* < 0.05 for trend for total stroke stratified by sex; *P* < 0.05 for trend for total stroke in people with hypertension and for fatal stroke in people without hypertension; Supplementary Table 1).

We also assessed the association between stroke outcomes and average glucose levels among participants without diabetes (Table 3). For an average glucose level higher than 140 mg per deciliter compared with 90–99.9 mg per deciliter, the adjusted HRs for total stroke and fatal stroke were 3.66 (95% CI, 1.47–9.08) and 5 (95% CI, 1.77–14.15), respectively.

Associations between glucose variability and the development of stroke are presented in Table 4. Among participants, an increased risk of nonfatal stroke with increasing glucose SD was significant (*P*=0.0198) for model 3. For a glucose SD level higher than 13.83 mg per deciliter, compared with less than 5.91 mg per deciliter, the adjusted HR for total stroke was 2.31 (95% CI, 1.19–4.48).

## 4. Discussion

In this prospective cohort study including 12,321 Chinese adults, we found that higher FPG levels increased the risk of stroke after controlling for other potential confounders. However, an association between elevations in FPG in the nondiabetic range and long-term risk of stroke outcomes was not found in our study. Furthermore, variation in FPG measurements was strongly correlated with stroke outcomes, which indicates that therapies should be evaluated further to minimize glucose fluctuation in diabetic patients to prevent stroke outcomes.

Diabetes is an established independent risk factor for stroke [14]. Previous studies have demonstrated that a high risk of myocardial infarction and other macrovascular diseases can be predicted by long-term glycemia [5, 8]. However, the stroke risk related to glycemia below the current diabetic threshold remains controversial. Some prior studies have reported a positive association between elevated FPG levels and stroke [15], while many others showed opposite conclusions [16]. A study of 47,865 men aged 34–54 years from the Aerobics Center Longitudinal Study found that, in FPG levels of 110 mg/dL or higher, each 10-unit increment of FPG was associated with a 6% higher risk of total stroke events. However, they had just one baseline measurement of FPG, and the cohort population may have reduced the possibility that many confounders had influence. In a prospective cohort study of 3,246 British women aged 60–79 years old, there was no evidence of linear or nonlinear associations between either fasting glucose or HbA1c and incident stroke, suggesting that in 60–79-year-old women [17], insulin resistance, rather than chronic hyperglycemia, is a more important risk factor for stroke. In our study, average glucose levels over 126–139.9 mg/dl may be risk factors for stroke according to all the participants; however, for undiagnosed group, FPG had little if any association with the risk of stroke, which only clearly increased at FPG ≥140 mg/dl after adjusting for classic risk factors. These inconsistent findings may be due to differences in study populations, length of follow-up, stroke outcome definitions (such as fatal, nonfatal, or a combination), confounder selection, or a combination of these factors.

In addition, blood glucose variation may also be related to the risk of stroke. A study in Taiwan reported that variation in FPG measurements can predict stroke in type 2 diabetes patients [18], showing that the coefficient of variation of FPG (FPG-CV) is a glucose variation measure that pinpoints the association between oscillating plasma glucose and stroke, independent of HbA1c. However, glycemic variation as an independent predictor of stroke among participants without diabetes remains incompletely defined. We investigated the standard deviation (SD) of long-term FPG variability during the 5-year follow-up and observed a novel predictor representing glucose instability (SD), indicating higher risk of the development of fatal stroke outcomes. Thus, the evaluation of the effects of long-term variation in blood glucose levels may be highly significant for stroke prevention.

The major hazards of hyperglycemia were specifically due to the irreversible damage caused by high glucose levels. Hyperglycemia can cause related diseases by damaging vascular endothelial cells in the central nervous system [19]. At least 4 major pathways are involved in hyperglycemia-induced vascular damage. Deleterious metabolic events are thought to be triggered by a single process: the overproduction of superoxide by the mitochondrial electron-transport chain indicates that the activation of oxidative stress by hyperglycemia plays a major role in the pathogenesis of diabetic complications [20]. This overproduction can result in endothelial dysfunction and contribute to vascular damage. Evaluated glucose levels have now been seen as a time-varying phenomenon. However, most previous studies merely used a single measure of FPG to evaluate the association between hyperglycemia and stroke outcomes, failing to take into account the long-term effects in FPG concentrations over time and neglecting repeated obtained measurements of random fasting blood glucose and glycated hemoglobin. Recent studies have reported that glucose fluctuations during glucose swings exhibited a more specific triggering effect on oxidative stress than chronic sustained hyperglycemia [21, 22]. Some studies reported that acute glucose fluctuations can enhance brain microvascular endothelial barrier dysfunction, which may implicate cerebral microvasculature [23]. Acute fluctuations also influence endothelial function, even in nondiabetic subjects and play a significant role in the risk of several chronicle complications [24–27]. The findings indicate that future therapy for DM should not only target the chronic sustained hyperglycemia index, such as fasting glucose levels and HbA1c, but also control glycemic variability in clinical practice. Future studies should pay attention to the verification of whether the risk of stroke outcomes can be controlled, or even reduced, after stabilizing glucose levels.

The strengths of our study include its prospective community-based design and the selection of an analytical strategy called the hierarchical Bayesian framework to assess the average FPG, making full use of all measured blood glucose values to estimate the average blood glucose load during follow-up as accurately as possible and develop a time-varying estimate of glucose levels. This analytical approach enabled us to incorporate clinically obtained measurements of random fasting blood glucose and HbA1c in a single composite estimate of glucose exposure and combine information on glucose levels from these measures, while stabilizing estimates for individuals with relatively sparse observations, by borrowing information across participants, and accounting for the relative variability in glucose and HbA1c measures to create our combined estimate.

Several limitations should be acknowledged. First, the possibility of confounding by unmeasured or unknown factors cannot be excluded. Some participants may have reached the criteria for diabetes but were allocated into the undiagnosed diabetes group because they did not present clinical symptoms after enrollment, which may be a cause of bias. However, considering that DM is a chronic disease, once the participants were diagnosed during a five-year follow-up, they were assigned to a DM group. Second, the assessment of glucose variability is complex, and only analyzing the long-term FPG levels may overlook the potential effects of postprandial blood glucose levels and some other factors, which may result in a lack of representation [28, 29]. Third, we were unable to consider stroke subtypes and failed to analyze the possibility of differences in outcomes between several stroke subtypes. Additionally, although we have recorded participants' medication status when they enrolled, there was no detailed information on the antihypertensive or lipid-lowering drug, which may be impact factors associated with blood glucose and stroke risk.

In conclusion, we observed that higher FPG levels are associated with an increased risk of stroke incident. Moreover, the variability in long-term FPG is closely correlated with the risk of fatal stroke outcomes. Our findings have important public health value and are highly significant for guiding clinical treatment for diabetes.

## Figures and Tables

**Table 1:** Baseline characteristics of the study participants^*∗*^.

	Total (*N* = 12,321)	Participants without diabetes (*N* = 11,310)	Participants with diabetes (*N* = 1,011)	*P* value
Age	60.8 ± 10.2	60.8 ± 10.3	61.3 ± 9.6	0.1173
Female sex	6,614 (53.7)	6,073 (53.7)	541 (53.51)	0.9103
BMI	23.6 ± 3.1	23.5 ± 3.1	24.8 ± 3.3	<.0001
Systolic blood pressure (mmHg)	141 ± 20.4	140.4 ± 20.2	148.2 ± 20.5	<0.0001
Diastolic blood pressure (mmHg)	84.1 ± 12.3	83.8 ± 12.3	86.7 ± 12.4	<0.0001
Fasting plasma glucose (mg/dL)	106.7 ± 21.9	102.5 ± 11.5	154 ± 43.8	<0.0001
Total cholesterol (mg/dL)	185.1 ± 34.3	184.7 ± 33.8	189.7 ± 39	<0.0001
LDL-C (mg/dL)	100.6 ± 26.7	100.3 ± 26.5	103.9 ± 28.3	<0.0001
HDL-C (mg/dL)	57.9 ± 15.6	58.3 ± 15.6	53.8 ± 14.6	<0.0001
Triglycerides (mg/dL)	120.3 ± 85.8	117.5 ± 81.5	152.3 ± 119.7	<0.0001
Education level (no formal education)	6,096 (49.5)	5,605 (49.6)	491 (48.6)	0.3049
Smoke				0.4424
Never smoked	8,284 (67.2)	7,590 (67.1)	694 (68.6)	
Current smoker	3,056 (24.8)	2,822 (24.9)	234 (23.2)	
Former smoker	981 (8)	898 (7.9)	83 (8.2)	
Alcohol drinking	2,801 (22.7)	2,578 (22.8)	223 (22.1)	0.5924
Regular exercise	5,332 (43.3)	4,849 (42.9)	483 (47.8)	0.0026
Hypertension	6,339 (51.4)	5,625 (49.7)	714 (70.6)	<0.0001
High cholesterol	3,712 (30.1)	3,350 (29.6)	362 (35.8)	<0.0001

^*∗*^Continuous variables are expressed as mean ± SD or as median (interquartile range). Categorical variables are expressed as frequency (percentage).

**Table 2:** Risk of incident stroke associated with average glucose level (*N* = 12,321).

	Average glucose level (mg/dl)						*P* for trend
	80–89.9	90–99.9	100–109.9	110–125.9	126–139.9	140-	
Total stroke							
Events, *n* (%)	5 (2.07)	97 (1.90)	105 (2.18)	30 (2.94)	27 (3.55)	15 (4.01)	
Model 1	0.73 (0.32–1.68)	1	1.12 (0.85–1.48)	1.46 (0.97–2.20)	1.99 (1.30–3.05)	2.31 (1.34–3.98)	0.0007
Model 2	0.70 (0.31–1.61)	1	1.18 (0.9–1.55)	1.40 (0.92–2.11)	1.98 (1.29–3.03)	2.18 (1.26–3.77)	<0.0001
Model 3	0.74 (0.32–1.69)	1	1.11 (0.84–1.46)	1.29 (0.85–1.95)	1.78 (1.16–2.75)	1.89 (1.09–3.29)	0.0009
Nonfatal stroke							
Events, *n* (%)	3 (1.24)	42 (0.82)	49 (1.02)	17 (1.67)	15 (1.97)	7 (1.87)	
Model 1	1.03 (0.31–3.22)	1	1.24 (0.82–1.86)	1.73 (0.98–3.04)	2.38 (1.32–4.29)	2.23 (1.01–4.97)	0.0008
Model 2	1.01 (0.31–3.28)	1	1.25 (0.83–1.99)	1.75 (1.00–3.09)	2.41 (1.33–4.34)	2.19 (0.98–4.88)	0.0009
Model 3	1.06 (0.33–3.42)	1	1.17 (0.77–1.78)	1.60 (0.90–2.82)	2.16 (1.19–3.93)	1.87 (0.83–4.22)	0.0067
Fatal stroke							
Events, *n* (%)	2 (0.83)	55 (1.08)	56 (1.16)	13 (1.27)	12 (1.58)	8 (2.14)	
Model 1	0.53 (0.13–2.16)	1	1.02 (0.7–1.48)	1.22 (0.66–2.23)	1.64 (0.88–3.06)	2.4 (1.14–5.04)	0.0096
Model 2	0.42 (0.1–1.74)	1	1.09 (0.75–1.58)	1.05 (0.57–1.94)	1.5 (0.8–2.8)	2.13 (1.01–4.5)	0.0245
Model 3	0.43 (0.11–1.79)	1	1.02 (0.7–1.49)	0.98 (0.53–1.81)	1.34 (0.71–2.54)	1.85 (0.86–3.96)	0.0871

Model 2, adjusted for age, sex; Model 3, adjusted for age, sex, education, smoke, drink, sport, BMI, systolic blood pressure, total cholesterol.

**Table 3:** Risk of incident stroke associated with average glucose level over the preceding 5 years among participants without diabete (*N* = 11,310).

	Average glucose level (mg/dl)						*P* for trend
	80–89.9	90–99.9	100–109.9	110–125.9	126–139.9	140-	
Total stroke							
Events, *n* (%)	5 (2.07)	96 (1.88)	104 (2.18)	27 (3.21)	7 (2.33)	5 (8.20)	
Model 1	0.74 (0.3–1.83)	1	1.11 (0.84–1.47)	1.53 (1–2.35)	1.27 (0.59–2.74)	4.24 (1.72–10.44)	0.0050
Model 2	0.68 (0.28–1.67)	1	1.17 (0.88–1.54)	1.49 (0.97–2.3)	1.25 (0.58–2.69)	4.07 (1.65–10.05)	0.0045
Model 3	0.7 (0.28–1.73)	1	1.1 (0.83–1.46)	1.39 (0.9–2.14)	1.15 (0.53–2.5)	3.66 (1.47–9.08)	0.0205
Nonfatal stroke							
Events, *n* (%)	3 (1.24)	42 (0.82)	49 (1.03)	15 (1.78)	4 (1.33)	1 (1.64)	
Model 1	0.84 (0.26–2.72)	1	1.25 (0.83–1.88)	1.78 (0.99–3.21)	1.44 (0.52–4.03)	1.56 (0.21–11.33)	0.0586
Model 2	0.88 (0.27–2.85)	1	1.30 (0.86–1.96)	1.84 (1.02–3.33)	1.46 (0.52–4.07)	1.61 (0.22–11.70)	0.0475
Model 3	0.90 (0.28–2.91)	1	1.24 (0.82–1.88)	1.72 (0.95–3.13)	1.38 (0.49–3.87)	1.53 (0.21–11.22)	0.0912
Fatal stroke							
Events, *n* (%)	2 (0.83)	54 (0.83)	55 (1.15)	12 (1.43)	3 (1.00)	4 (6.56)	
Model 1	0.54 (0.13–2.23)	1	1.02 (0.7–1.49)	1.3 (0.69–2.43)	1.09 (0.34–3.51)	7.19 (2.59–19.98)	0.0345
Model 2	0.43 (0.11–1.78)	1	1.09 (0.75–1.59)	1.14 (0.6–2.14)	0.99 (0.31–3.19)	5.92 (2.12–16.59)	0.0481
Model 3	0.45 (0.11–1.85)	1	1.01 (0.69–1.49)	1.06 (0.56–2)	0.88 (0.27–2.85)	5 (1.77–14.15)	0.1295

Model 2, adjusted for age, sex; Model 3, adjusted for age, sex, education, smoke, drink, sport, BMI, systolic blood pressure, total cholesterol.

**Table 4:** Risk of incident stroke associated with variance (*N* = 11,310).

	Standard deviation				*P* for trend
	Quartile 1 (<5.91)	Quartile 1 (5.91 to 8.9)	Quartile 1 (8.91 to 13.83)	Quartile 1 (>13.83)	
Total stroke					
Events, *n* (%)	42 (1.68)	48 (1.92)	40 (1.60)	75 (3.00)	
HR	1	1.25 (0.85–1.84)	1.72 (1.15–2.59)	1.83 (1.07–3.11)	0.001
Nonfatal stroke					
Events, *n* (%)	16 (0.64)	33 (1.32)	26 (1.04)	40 (1.6)	
HR	1	1.47 (0.87–2.47)	1.98 (1.14–3.43)	1.71 (0.79–3.72)	0.008
Fatal stroke					
Events, *n* (%)	26 (1.04)	15 (0.60)	14 (0.56)	35 (1.40)	
HR	1	1.13 (0.66–1.95)	1.34 (0.74–2.45)	2.31 (1.19–4.48)	0.0198
Participants without diabetes					
Total stroke					
Events, *n* (%)	42 (1.7)	47 (1.9)	39 (1.63)	46 (2.56)	
HR	1	1.33 (0.89–2.00)	1.11 (0.52–2.36)	3.49 (1.43–8.55)	0.0246
Nonfatal stroke					
Events, *n* (%)	26 (1.05)	32 (1.3)	25 (1.04)	23 (1.28)	
HR	1	1.56 (0.90–2.70)	1.25 (0.46–3.41)	1.42 (0.2–10.23)	0.2053
Fatal stroke					
Events, *n* (%)	16 (0.65)	15 (0.60)	14 (0.58)	23 (1.28)	
HR	1	1.24 (0.70–2.17)	0.90 (0.28–2.85)	6.38 (2.55–15.95)	0.016

Adjusted for age, sex, education, smoke, drink, sport, BMI, systolic blood pressure, total cholesterol.

## Data Availability

Data are available in a public, open-access repository (http://charls.pku.edu.cn/zh-CN/page/data/2011-charls-wave1; http://charls.pku.edu.cn/zh-CN/page/data/2013-charls-wave2; http://charls.pku.edu.cn/zh-CN/page/data/2015-charls-wave4).

## Supplementary Materials

Supplementary Materials Figure S1: number of times of participants blood glucose measurements. Figure S2: number of times of participants glycated hemoglobin A1c measurements. Supplementary Table 1: risk of incident stroke associated with average glucose level stratification by sex or hypertension.
