# Supplementary material for: Longitudinal Average Glucose Levels and Variance and Risk of Stroke: A Chinese Cohort Study
Source: Int J Hypertens. 2020 Apr 21;2020:8953058. doi: 10.1155/2020/8953058 (PMC7191433; doi:10.1155/2020/8953058)
Supplement: Supplementary Materials — Figure S1: number of times of participants blood glucose measurements. Figure S2: number of times of participants glycated hemoglobin A1c measurements. Supplementary Table 1: risk of incident stroke associated with average glucose level stratification by sex or hypertension. [file 8953058.f1.pdf]

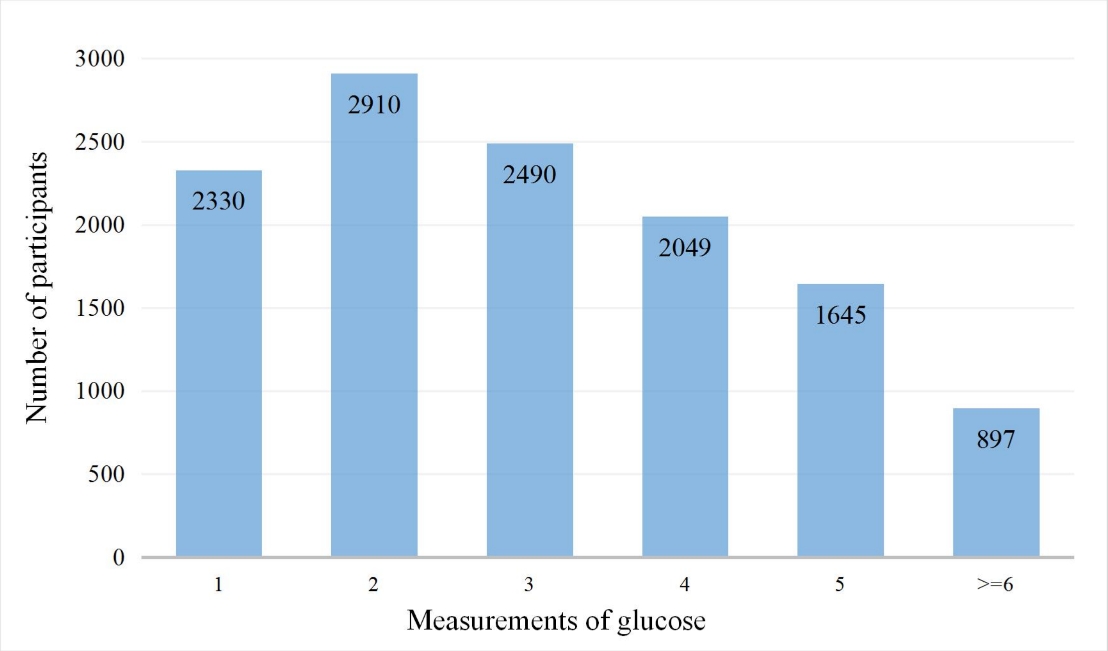


Figure S1 Number of times of participants blood glucose measurements*

* 40,975 clinical measurements of glucose levels from 12,321 participants without stroke were used to examine the relationship between glucose levels and the risk of stroke. Specific number of times of participants blood glucose measurements are shown in this figure.

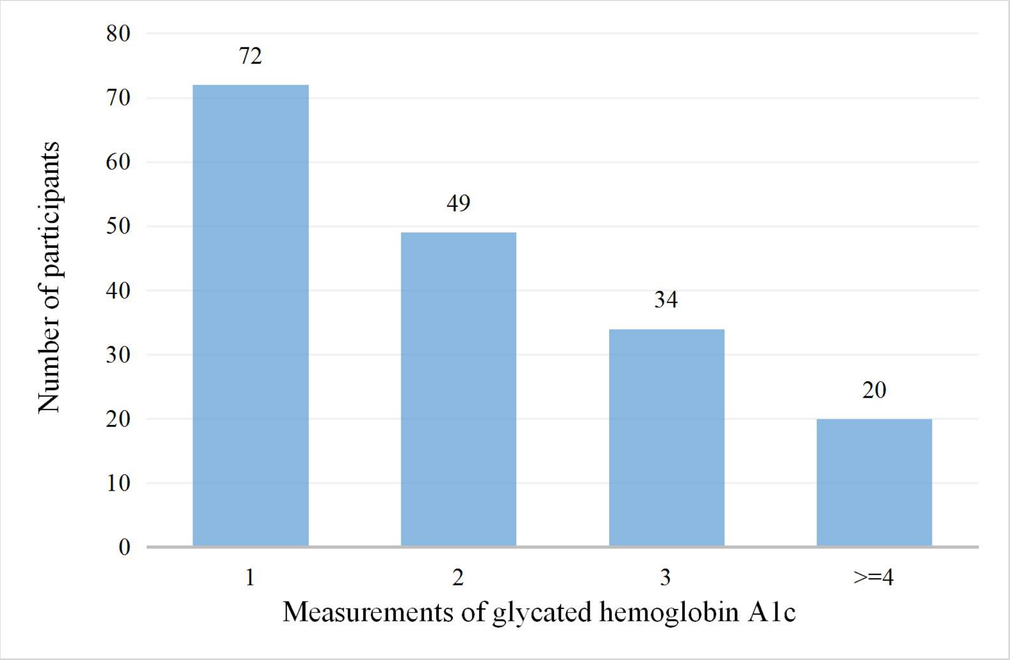


Figure S2 Number of times of participants glycated hemoglobin A1c measurements*

* 367 measurements of glycated hemoglobin A1c levels from 12,321 participants without stroke were used to examine the relationship between glucose levels and the risk of stroke. Specific number of times of participants glycated hemoglobin A1c measurements are shown in this figure.

Supplementary Table 1 Risk of Incident Stroke Associated with Average Glucose Level Stratification by sex or hypertension

| | Average Glucose Level (mg/dl) | | | | | | |
|---|---|---|---|---|---|---|---|
| | 80-89.9 | 90-99.9 | 100-109.9 | 110-125.9 | 126-139.9 | 140- | P for trend |
| Male | | | | | | | |
| Total stroke | 0.88 (0.32-2.45) | 1 | 1.06 (0.7-1.59) | 1.56 (0.85-2.85) | 2.05 (1.12-3.76) | 1.28 (0.45-3.59) | 0.0313 |
| Nonfatal stroke | 1.02 (0.24-4.32) | 1 | 1.15 (0.66-2) | 1.75 (0.81-3.79) | 1.56 (0.63-3.84) | 1.01 (0.23-4.37) | 0.2914 |
| Fatal stroke | 0.71 (0.17-3.01) | 1 | 1 (0.54-1.83) | 1.3 (0.49-3.46) | 2.46 (1.07-5.61) | 1.73 (0.4-7.48) | 0.0472 |
| Female | | | | | | | |
| Total stroke | 0.39 (0.05-2.84) | 1 | 1.12 (0.76-1.66) | 1.16 (0.65-2.06) | 1.58 (0.85-2.97) | 2.28 (1.16-4.48) | 0.0102 |
| Nonfatal stroke | 1.09 (0.14-8.26) | 1 | 1.25 (0.66-2.35) | 1.55 (0.66-3.64) | 3.04 (1.33-6.92) | 2.86 (1.03-7.89) | 0.0041 |
| Fatal stroke | None* | 1 | 1.06 (0.64-1.74) | 0.88 (0.4-1.96) | 0.71 (0.25-2.06) | 1.87 (0.75-4.66) | 0.4569 |
| With hypertension | | | | | | | |
| Total stroke | 0.55 (0.14-2.27) | 1 | 1.07 (0.77-1.49) | 1.16 (0.72-1.87) | 1.5 (0.9-2.5) | 1.7 (0.92-3.11) | 0.0257 |
| Nonfatal stroke | 0.64 (0.09-4.72) | 1 | 1.16 (0.72-1.9) | 1.59 (0.84-3) | 1.94 (0.98-3.86) | 2.04 (0.88-4.73) | 0.0118 |
| Fatal stroke | 0.47 (0.06-3.42) | 1 | 0.99 (0.63-1.55) | 0.78 (0.37-1.63) | 1.05 (0.48-2.29) | 1.32 (0.55-3.21) | 0.6739 |
| Without hypertension | | | | | | | |
| Total stroke | 0.99 (0.3-3.26) | 1 | 0.92 (0.52-1.62) | 1.33 (0.54-3.22) | 2.3 (1-5.28) | 1.74 (0.41-7.44) | 0.0974 |
| Nonfatal stroke | 1.92 (0.43-8.54) | 1 | 0.84 (0.36-1.96) | 0.85 (0.19-3.81) | 2 (0.57-7.03) | None | 0.7887 |
| Fatal stroke | 0.45 (0.06-3.43) | 1 | 1.05 (0.49-2.25) | 1.7 (0.55-5.2) | 2.5 (0.82-7.63) | 5.06 (1.1-23.31) | 0.0133 |

Subgroup analyses of the association between average glucose level and the risk of incident stroke. Hazard ratios (HRs) and the 95% confidence interval in all participants stratified by sex, hypertension were calculated by Cox regression model after adjustment for age, education, smoke, drink, sport, BMI, total cholesterol. Hypertension was defined as systolic or diastolic blood pressure of 140/90mmHg or higher or a history of hypertension.

*None: without adverse events
